# Increased prevalence of synaesthesia in musicians

**DOI:** 10.1177/03010066251390106

**Published:** 2025-10-29

**Authors:** Linden Williamson, Scott Bailey, Jamie Ward

**Affiliations:** 1 Department of Psychology, 7302 Texas Lutheran University, Seguin, TX, USA; 2 School of Psychology, 1948 University of Sussex, Brighton, UK

**Keywords:** Synaesthesia/synesthesia, music, cross-modal, expertise, creativity

## Abstract

Although synaesthesia has been linked to increased creativity and engagement with the arts, most of the evidence has come from visual arts rather than music. Here we show for the first time that synaesthesia is far more prevalent in musicians than non-musicians (an odds ratio of about 4). We show that this result holds true for all three different kinds of synaesthesia that we considered (grapheme-colour, sequence-space, sound-colour) including for types of synaesthesia unrelated to music. That is, it is not simply the case that the ability to ‘see’ music drives the higher prevalence, although this may have a role. Instead, we speculate that the cognitive profile of synaesthetes is conducive to musicality. We provide an estimate of the prevalence of sound-colour synaesthesia in non-musicians of between 0.3% and 1.3%, depending on the threshold applied, with comparable figures for musicians of 1.3% to 7.3%.

## Introduction

Synaesthesia is an extraordinary way of experiencing the world in which music can be seen (as well as heard), numbers can be coloured, and words can have tastes. Various defining features of synaesthesia have been proposed such as automaticity, consistency, and idiosyncrasy (e.g., Cytowic, 1989; Simner, 2012; Ward, 2019) of which the most commonly agreed criterion is that it requires the pairing of an eliciting stimulus (termed inducer) with the synaesthetic experience itself (termed concurrent) (Grossenbacher & Lovelace, 2001). These experiences emerge early and persist across the lifespan (Meier et al., 2014; Simner & Bain, 2013). It runs in families (e.g., Asher et al., 2009) and is linked to differences in brain structure and function (Rouw et al., 2011). One account for why synaesthesia persists in the population is that it is linked to certain beneficial traits such as creative cognition (Brang & Ramachandran, 2011). Indeed, synaesthesia has been linked to increased engagement in the creative arts in terms of hobbies and occupations (Lunke & Meier, 2022; Rich et al., 2005; Ward et al., 2008), and choice of degree subject (Rothen & Meier, 2010). It has also been linked to better performance on psychometric measures of creativity (Chun & Hupe, 2016; Ward et al., 2008) as measured on tasks of divergent thinking (e.g., finding an alternate use for a common object) and convergent thinking (e.g., finding a common link between different words). It is also linked to higher scores on personality traits such as openness to experience which are linked to creative tendencies (Banissy et al., 2013; Rouw & Scholte, 2016). A similar profile is found in musicians (Gibson et al., 2009). However, there are key gaps in our understanding of whether different forms of creative expression (e.g., visual art versus music) are linked to different profiles of synaesthesia (e.g., whether specific to certain inducers or concurrents). This study addresses this question with a particular focus on musicianship. Our primary hypothesis is that there will be an increased prevalence of synaesthesia in musicians. Our secondary hypothesis is that this association (between musicianship and synaesthesia) will be greater when music is itself an inducer of synaesthesia.

There are two broad approaches for establishing a link between synaesthesia and creative engagement. One is to take a group of synaesthetes (versus controls) and assess different forms of creative engagement and expertise. The complementary approach is to take groups of experts (e.g., artists versus scientists; musicians versus non-musicians) and assess the prevalence of synaesthesia. We consider these approaches in turn. Rich et al. (2005) examined occupations, hobbies, and self-reported strengths-weaknesses in a sample of synaesthetes who predominantly experienced colours to letters, numbers, and/or words. Significant differences, relative to non-synaesthetes, were found for art (as an active hobby and strength) but no differences were found for music. Ward et al. (2008) asked a heterogeneous group of synaesthetes about the amount of time spent producing visual art and playing a musical instrument. Both were significantly higher in synaesthetes relative to controls. Engagement with music was also significantly higher for synaesthetes for whom music and sound acted as an inducer of visual experiences relative to other synaesthetes (engagement with visual art was not modulated in this way). With regard to the second approach, Rothen and Meier (2010) examined the prevalence of grapheme-colour synaesthesia in students engaged in a visual arts degree versus science programmes. The prevalence in these two groups was 7.1% and 2.1%, respectively (an odds ratio of 3.6). A comparable study of musicians has not been performed (although there is research on the characteristics of synaesthetic musicians; Glasser, 2021; Glasser, 2023). The current study uses a comparable approach to Rothen and Meier (2010) with a focus on musicians instead of artists and with a key difference of considering the prevalence of several types of synaesthesia. There is also an additional consideration of engagement with visual art.

A consideration of types of synaesthesia is important because it could be the case that different types of synaesthesia are linked to different skillsets and different forms of creative engagement. For example, those with colour experiences might gravitate towards visual arts, those with music-induced experiences gravitate to music, those with mirror-touch (who feel, on their own body, observed sensations on other people) to dance or sculpture. The alternative view is that synaesthesia itself is linked to some generic differences in cognitive function (e.g., in memory, imagery, perception) that transcend the idiosyncratic ways that it can be manifested. For example, this may occur because synaesthetes have a distinctive brain connectivity profile that affects cognition beyond synaesthesia (Brang & Ramachandran, 2011) put simply, additional connections in the brains of synaesthetes may also enable them to generate novel conceptual associations that underpin creative thinking. Indeed, there is evidence for both positions. A recent study gave a questionnaire measure of musicality, called the GOLD-MSI (Müllensiefen et al., 2014), to two groups of synaesthetes (those for whom music and other sounds was an inducer of visual experiences, and those for whom it was not) and a control group of non-synaesthetes (Ward et al., 2024). In general, both groups of synaesthetes reported high levels of musicality across measures of perception (e.g., detecting out of tune notes) and production (e.g., singing). Other research, using more objective measures, corroborates the finding that synaesthesia is linked to differences in auditory perception (Del Rio et al., 2024; Ward et al., 2021) and musical memory (Lunke & Meier, 2018; Mealor et al., 2020) that transcends how it is manifested as different types. However, the Ward et al. (2024) study found that in addition to differences in self-reported musicality between synaesthetes and non-synaesthetes there were also differences within the synaesthetes. Those with sound-colour reported higher levels of musicality. Overall differences linked to the presence of synaesthesia together with more specific differences linked to the number or nature of types was also found for measures of artistic engagement, including music, by Lunke and Meier (2022).

As a note on terminology, we use the term sound-colour synaesthesia to denote people for whom a wide range of sounds (including music) trigger colours. This is also termed chromaesthesia. There is known heterogeneity within this group in terms of what acoustical, musical, or semantic features within a sound may drive the association (e.g., pitch, note names, timbre) (e.g., Curwen, 2022; Itoh et al., 2017). For some synaesthetes music, but not other sounds, can trigger synaesthesia (e.g., linked to musical keys such as F-minor; Haack & Radocy, 1981). These finer-grained distinctions are not explored here and those who we refer to as sound-colour synaesthetes have passed a test in which single musical tones are associated with colour. The experiences of these synaesthetes are typically complex and extend beyond colour, unfolding over time. For example, one synaesthete described a Brahms Intermezzo as coloured pictures that she called ``maps’’ that correspond to different sections or themes of the music either as blocks of colour, or numerous coloured lines going in different directions (Mills et al., 2003).

The present study examines the prevalence of synaesthesia in musicians and non-musicians, defined primarily through professional engagement (working in the music industry). The prevalence of three different types of synaesthesia is assessed using ‘gold standard’ measures of test–retest consistency for: grapheme-colour, music tone-colour, and sequence-space (where the latter refers to the experience of sequential concepts such as numbers, days, months in a specific spatial pattern). Grapheme-colour and sequence-space were chosen as these are some of the most common types of synaesthesia that uses commonly used tests of synaesthesia (we would need a far larger sample to consider rarer variants such as lexical-gustatory). Tone-colour was chosen to be directly relevant to music, and due to the availability of a recently validated test (Ward et al., 2024). The prevalence of sound-colour synaesthesia is far less researched, but one study found 2 in 500 people (0.2% prevalence) (Simner et al., 2006). One challenge for assessing this type of synaesthesia is that consistency is a less reliable measure than for other forms of synaesthesia (e.g., because musical notes can have several colours, or non-synaesthetes can generate non-random sound-colour associations). In their recent study, Ward et al. (2024) addressed this by considering a set of four variables that are sensitive to this type of synaesthesia which includes consistency alongside others (synaesthetic colour palette, strong pitch-luminance associations, representational similarity between colour space and pitch or timbre). These are outlined in the method. Our primary hypothesis is that synaesthesia will be more prevalent in musicians, and our secondary aim is to determine whether this will be specific to sound-colour synaesthesia or reflect all kinds of synaesthesia (or something in between).

## Method

### Participants

**Figure 1.**
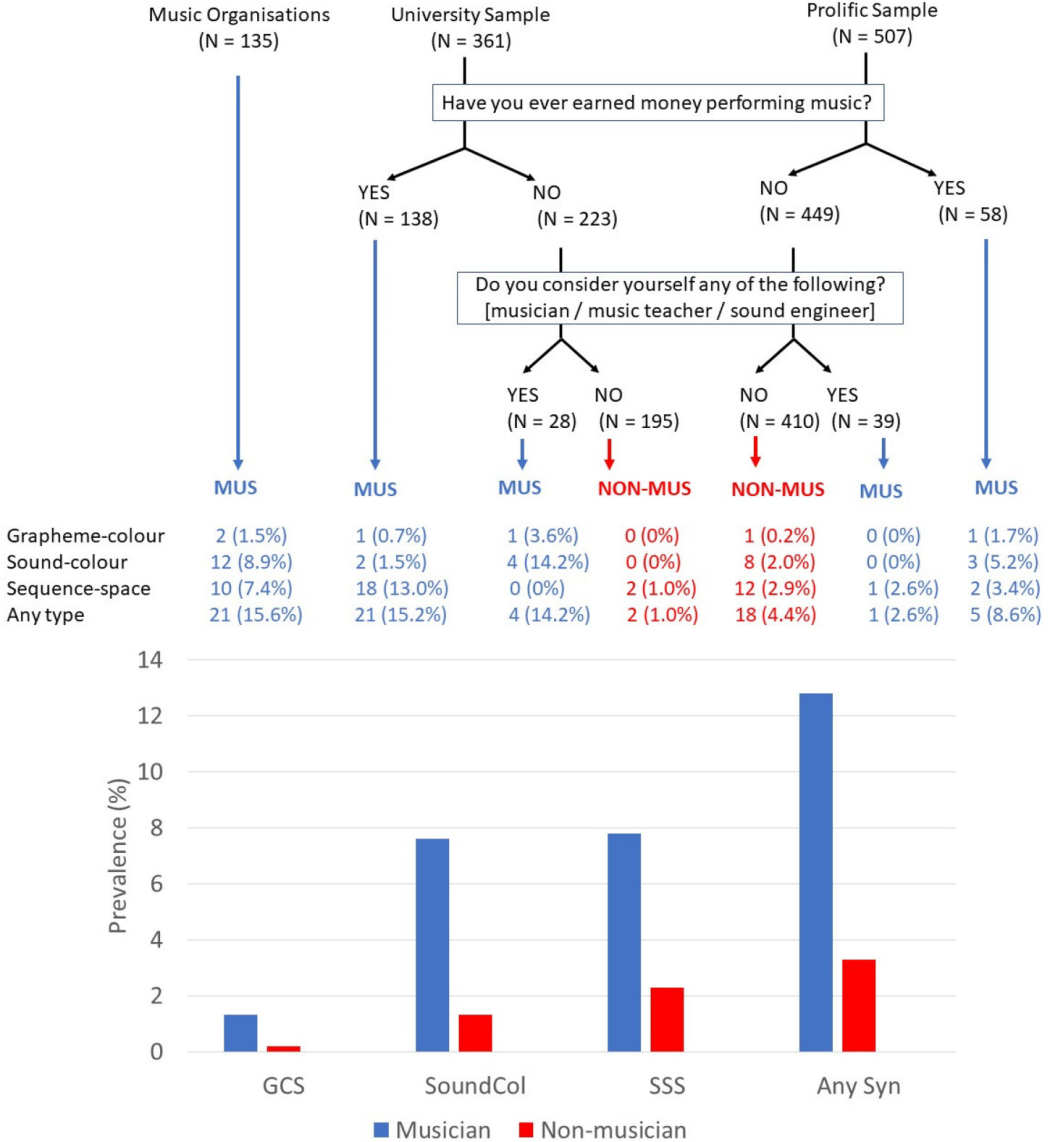
The prevalence of different forms of synaesthesia in musicians and non-musicians. Top: Results sub-divided according to the initial recruitment stream. Bottom: Results pooled across recruitment streams.

The combined sample, after exclusions, was 1003 participants. Participants were recruited from one of three sources: music organisations, a University sample, and an online general population sample (Prolific.co). The invitation to take part in research did not mention synaesthesia explicitly but participants were informed it was a two-part study in which some people would be selected to take part in a second online study. The music organisations Sonic Guild (formerly Black Fret, www.sonicguild.org/) and Orb (https://www.orbrecordingstudios.com/) represent leading artists in the Austin (Texas) region. Texas Lutheran University (TLU) is a liberal arts college based in the same region where the link was sent to both staff and students. Prolific is an online recruitment source which was chosen in order to recruit a more demographically representative US sample (i.e., not over-represented with musicians, a larger range of ages, etc.). The sample characteristics are shown in Table 1. We also created binary musician and non-musician groups collapsing across recruitment streams. For the university and prolific samples, status as a musician was determined by responses to two questions: ‘Have you ever earned money performing music?’ and ‘Do you consider yourself any of the following? [musician / music teacher / sound engineer]’. This is shown in Figure 1.

**Table 1.** Demographic characteristics of the three original samples, and the aggregated samples.

Original sample	N	Age, mean years (S.D.)	Gender
Music organisations	135	40.138 (12.584)	78 males, 47 women, 10 non-binary or prefer not to say
University staff & students	361	30.997 (14.845)	89 men, 265 women, 7 non-binary or prefer not to say
Prolific	507	44.055 (15.722)	242 men, 249 women, 16 non-binary or prefer not to say

Aggregated sample	N	Age, mean years (S.D.)	Gender
Musician	395	36.356 (15.020)	168 men, 214 women, 13 non-binary or prefer not to say
Non-musician	608	40.454 (16.709)	241 men, 347 women, 20 non-binary or prefer not to say

A post hoc power analysis (G*Power), based on the observed prevalence of all types of synaesthesia, produced an achieved power of 0.99 at alpha = 0.05. The study was approved by the Institutional Review Board of Texas Lutheran University.

### Materials and Procedure

Participants took part in up to two online sessions. The first was a brief survey run on Qualtrics (Provo, UT) which asked about hobbies, work in creative industries, and types of synaesthesia. Following an affirmative answer about synaesthesia, they were then directed to a second website (www.syntoolkit.org) where participants took up to three tests to validate different forms of synaesthesia (grapheme-colour, sound-colour, and sequence-space synaesthesia). These are described in turn below.

#### Questionnaire

The questions relating to hobbies, professions, and abilities analysed in this study were as follows:

- Do you consider yourself any of the following? Click as many as apply. [Actor/ Advertiser/ Artist/ Colour designer/ Dancer/ Decorator/ Florist/ Graphic illustrator/ Journalist/ Landscaper- gardener/ Musician/ Painter/ Music teacher/ Radio host/ Sound engineer/ Stagehand/ None of the above]
- Have you ever earned money doing visual art? [Yes / No]
- Have you ever earned money performing music? [Yes / No]

The questions asking about synaesthesia were preceded by a brief description of this condition as follows: ‘Synaesthesia is a rare trait of the brain that creates extra sensory experiences. Letters might have colours, or sounds might have tastes. These connections are formed early in childhood and do not change much with age. People with synaesthesia know exactly what their associations are, without having to think about it. They might know with certainty that the letter C is a specific shade of purple, and that this colour never really changes’. They were then asked the following six questions:

- Have you heard of synaesthesia before now? [Yes / No]
- Do letters or numbers cause you to have a colour experience? Example: Does the letter ‘J’ printed in black appear coloured to you? Does the number ‘5’ printed in black appear coloured to you? [Yes / No]
- Do weekdays and months have specific colours? Example: Does July always mean Navy Blue (or another colour) to you? Is Wednesday always orange (or another colour)? [Yes / No]
- Does hearing a sound make you perceive a colour? Example: Does a shrill car horn cause you to perceive a colour such as green? [Yes / No]
- Does listening to music make you experience colour, shape, texture, or a combination of the three? [Yes / No]
- Does thinking about time (months, days) or numbers always generate an image of that sequence? Example: the months of the year are arranged in a circle, or numbers appear in an oriented line. [Yes / No]

Participants who answered Yes to one or more of the synaesthesia questions were sent an additional link to tests of grapheme-colour synaesthesia, sequence-space synaesthesia, and sound-colour synaesthesia. Participants were directed to the tests considered relevant to themselves.

#### Grapheme-Colour Test

This is adapted from Eagleman et al. (2007). For people directed to this test, they were asked to indicate if their synaesthesia applied to letters or numbers or both. They were then given the following instructions: ‘The synaesthesia test will start on the next page. You will see numbers or letters on the screen next to a colour palette. For each number or letter, choose the colour you think it best goes with. You can choose any colour you like, but please don't pick the same colour for everything. Be as fast as you can’. The selection of stimuli depended on the answers to the initial question and consisted of 10 single digits (0–9) and/or 26 upper case letters (A-Z). There were three repetitions of each stimulus (so up to 48 trials), and participants either chose a specific colour or had the option of picking ‘no colour’. Participants had to generate colours for > 50% of trials to be analysed. For each grapheme, the difference in colour (in CIELuv space) is summed across the three repeated pairs, and then averaged. An average score of < 135 is indicative of having synaesthesia (Rothen et al., 2013).

#### Sequence-Space Synaesthesia

This test is taken from Ward et al. (2018). Participants are provided with the following instructions: ‘In this test you'll see numbers (0–9), days of the week (e.g., Tuesday), and months of the year (e.g., July) displayed in the centre of the screen. Your task is to think about how these concepts might be arranged spatially on a two-dimensional (i.e., flat) screen. Some people may automatically think about these concepts spatially in their everyday life, and if this is something you do, then you should use this. For other people this may seem like a strange task, but just go with your intuitions. There aren’t any right or wrong answers. When you see each item on the computer screen, visualise where it fits spatially and click the mouse in the corresponding location on the screen. Each item (number or day or month) is repeated three times’. They are then presented with 29 stimuli (7 weekdays, 12 months, 10 digits) which are written centrally on the screen and they make a mouse click to a spatial location on the screen. This is followed by a set of questions (e.g., ‘Before doing this experiment, I always thought about NUMBERS as existing in a particular spatial sequence’) and the answer to nine Likert questions are summed to give a score. Sequence-space synaesthesia is identified by a score of <= 19 on the questionnaire together with high consistency defined as mean differences in xy coordinates (across 3 repetitions of same stimulus) that are less than a cut-off score of < - 2 standard deviations away from randomly permuted xy coordinates (1000 repetitions) (Ward, 2022).

#### Sound-Colour Synaesthesia

This test is taken from Ward et al. (2024) and uses the same basic layout as for the grapheme-colour test (with a clickable icon to play the sound in place of a grapheme). There were 24 sounds and 3 repetitions (i.e., 72 trials). The sounds comprised three timbres (piano, string, pure tones) at eight pitch levels. Participants were first presented with these instructions: ‘in sound-colour synaesthesia sounds can produce an involuntary, automatic experience of colour but also shape or movement. In this task we are specifically interested in your associations between sound and colour. To do this task please use headphones if you have them, and make sure you are in a quiet and relaxed environment. This test involves listening to various sounds, so make sure that the volume is set to a comfortable level before you begin. The synaesthesia test will start on the next page. You will hear a series of sounds and we’d like to know if you experience any colours when you listen to them. Some people habitually and automatically associate colours with sounds. This is a type of synaesthesia. For those people, this will feel very natural. You may feel frustrated that you are struggling to choose an exact colour that matches your experience - just do your best. For other people this will feel like a strange task. That's ok. You can choose ‘no colour’ if you genuinely do not experience anything, and you can choose this option as many times as you like’.

People are classified as having sound-colour synaesthesia if they report a high number of colours to sounds (>50% of trials) together with two or more of the following indicative features after conversion from RGB to CIELuv: 1) high test–retest consistency with 3 repetitions (cut-off score of < 60.51, averaging across the 3 pairwise comparisons, indicative of synaesthesia), 2) a synaesthetic colour palette (cut-off score of p[syn] > .5 indicative of synaesthesia), 3) a strong pitch-luminance correspondence (cut-off score of > 31.196 indicative of synaesthesia), 4) strong representational similarity between sound features and colour space (cut-off score of > 0.322 indicative of synaesthesia); following Ward et al. (2024). This has a specificity of 0.85 and a sensitivity of 0.78.

## Results

Overall, we found N = 72 participants who passed one or more tests of synaesthesia. Synesthetes did not differ from non-synaesthetes in terms of either age (syns: 38.167 years [s.d. = 15.429], controls: 38.890 years, [s.d. = 16.245]; t(994)= 0.365, p = .715) or gender (syns 23:44:5 and controls 386:517:28 men: women: non-binary/other; χ^2^ (2) = 5.005, p = .082).

Figure 1 shows the prevalence of different types of synaesthesia divided according to their recruitment stream and also when pooled into musician versus non-musician groups (collapsing across recruitment stream). There is a significantly higher prevalence of synaesthesia in musicians considering sound-colour (χ^2^ (1) = 13.649, p < .001), grapheme-colour (χ^2^ (1) = 4.884, p = .027), sequence-space (χ^2^ (1) = 17.183, p < .001), and when pooling across all three of these types (χ^2^ (1) = 35.043, p < .001). These correspond to odds ratios of 4.211, 7.684, 3.576, and 4.396 respectively. Odds ratio is a measure of effect size calculated as follows:

(N mus + syn) / (N mus + nonsyn) ÷ (N nonmus + syn) / (N nonmus + nonsyn).

That is, synaesthesia is greatly over-represented in musicians and this occurs for types of synaesthesia in which music is an inducer and for types, such as sequence-space, that have no direct connection to music. Our design also enables us to consider different classifications for how we divide our sample into musician and non-musician. Taking a simple cut based solely on having earned money through music in the Prolific and University samples: we find a prevalence of synaesthesia of 13.3% in musicians (26/196) and 3.7% in non-musicians (25/672); χ^2^ (1) = 24.998, p < .001. This is similar to the 17.4% prevalence found in the sample of musicians recruited through music organisations. If we consider those people who have not earned money via music but self-identify as a musician (or closely related occupation) then the prevalence in these amateur musicians is 7.7% (5/65) compared to 3.3% (20/605) in non-musicians, and this does not reach significance (χ^2^ (1) = 2.910, p = .088) (A full breakdown by types of synaesthesia is reported in Figure 1 but the Ns here are too low to compare statistically).

**Figure 2.**
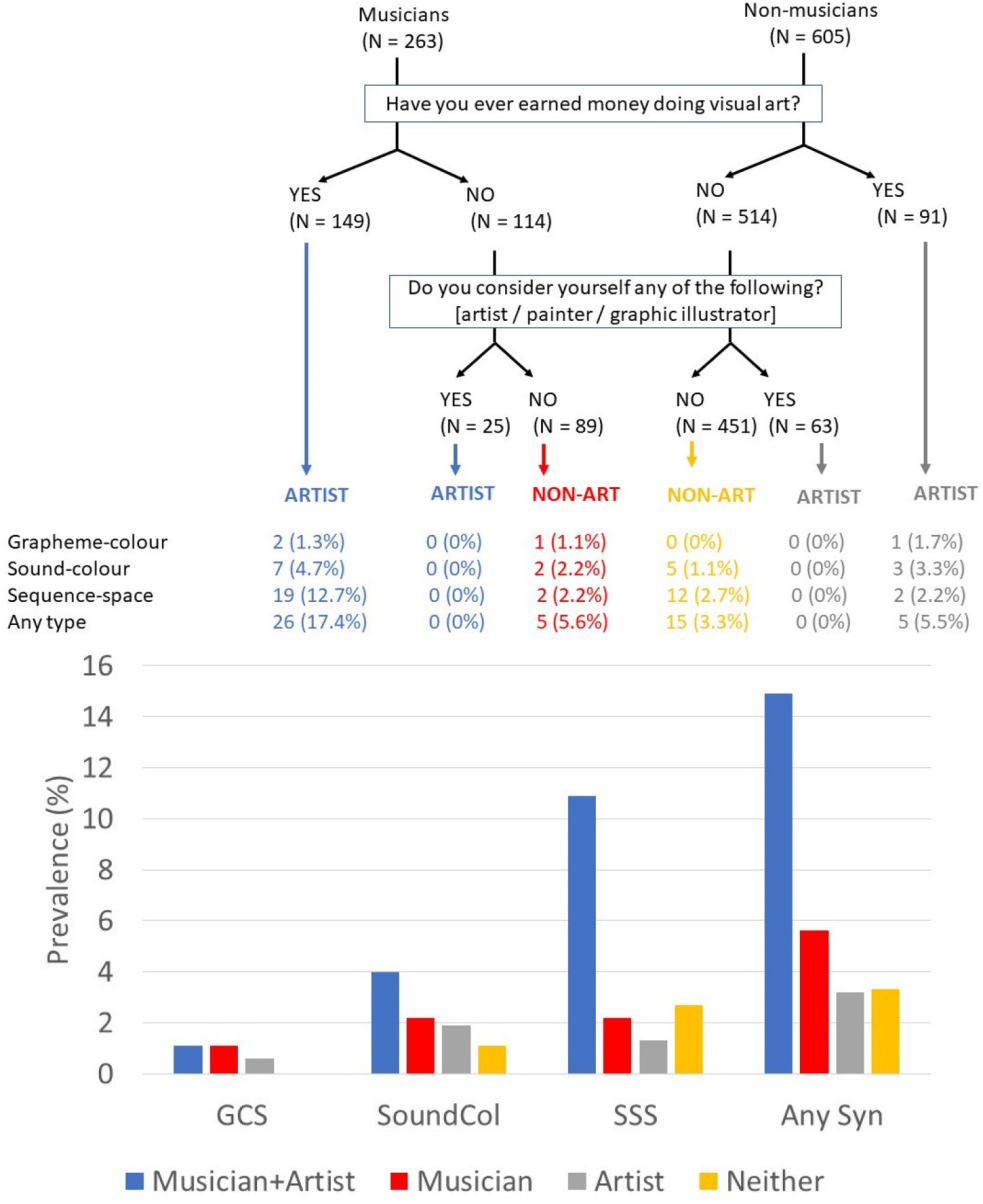
The prevalence of different forms of synaesthesia depending on musician status and level of engagement in visual art.

Considering next visual art engagement (which was asked about for the Prolific and University samples), we can divide participants according to whether or not they are active in visual arts as well as whether they are a musician or not. For this, we also considered responses to two questions: ‘Have you ever earned money doing visual art?’ and ‘Do you consider yourself any of the following? [artist / painter / graphic illustrator]’. The results are shown in Figure 2. Here we show that the prevalence of (all kinds of) synaesthesia is greatly increased in the group who are both musicians and artists (χ^2^ (1) = 30.344, p < .001, comparing prevalence of synaesthesia across the four groups).

**Figure 3.**
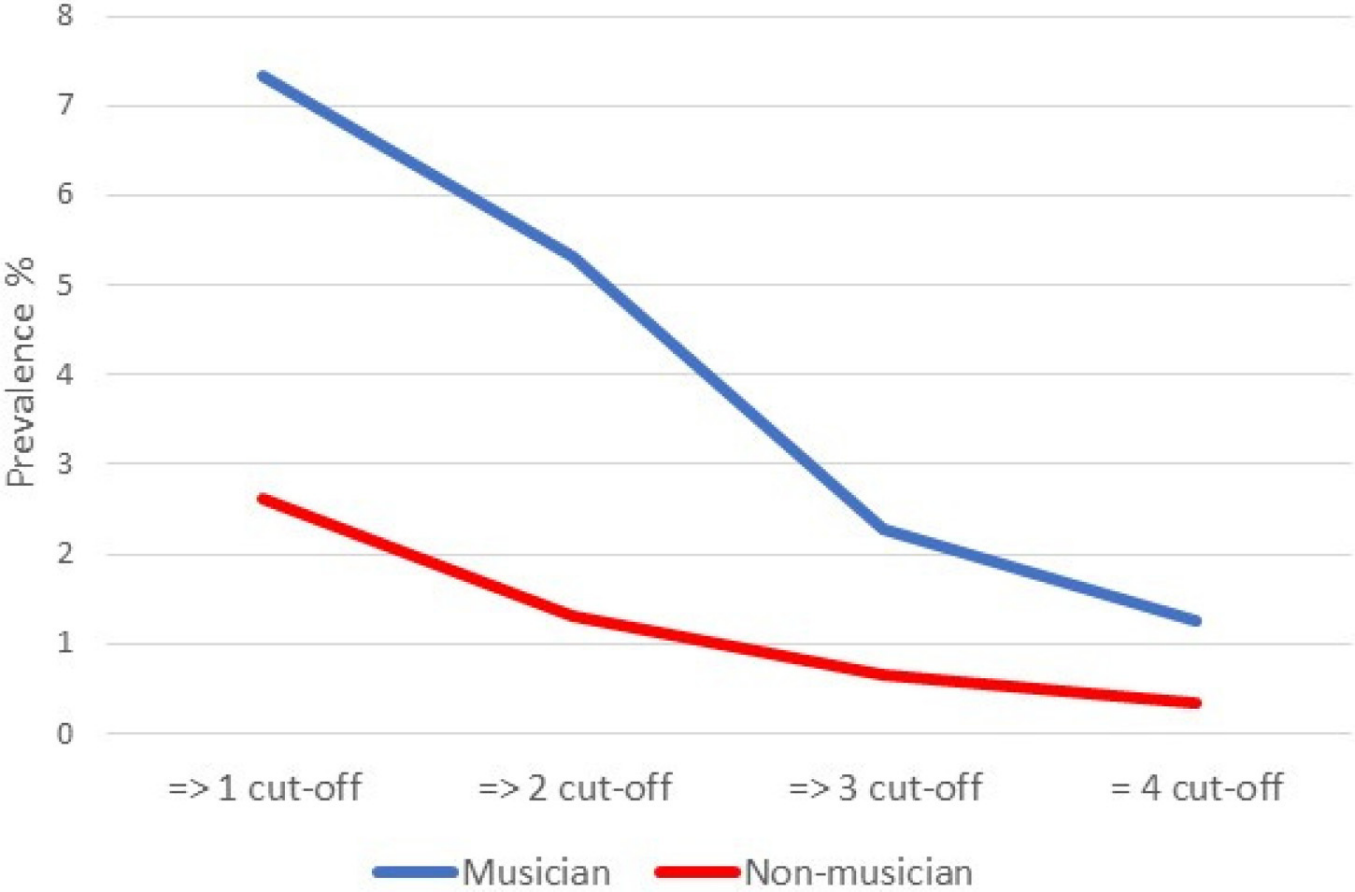
The prevalence (%) of music tone to colour synaesthesia in musicians and non-musicians as a function of the number of cut-off thresholds that need to be passed for having this type of synaesthesia.

Given the paucity of prior studies that have assessed the prevalence of sound-colour synaesthesia, we looked at this in more detail. Of note, our results contradict prior research (Simner et al., 2006) showing that grapheme-colour is more common than sound-colour (we found the opposite, even in non-musicians). However, prevalence estimates are directly related to the choice of threshold (liberal or conservative criteria for identifying synaesthesia) and the sensitivity/specificity of the test. The current choice of threshold (2 or more indicative features of sound-colour synaesthesia) was motivated by prior research (Ward et al., 2024) but Figure 3 shows how the prevalence changes as a result of more liberal or conservative thresholds. Importantly, our key finding of a prevalence difference amongst musicians and non-musicians is robust against different choices of threshold. At the a priori cut-off (2 or more features), the odds ratio is 4.211. At a more liberal threshold (>= 1) it is 2.932, and at more conservative thresholds it is 3.521 (>= 3) and 3.885 (=4). Notably, the prevalence in non-musicians at the most conservative threshold, 0.3%, is similar to the only existing prevalence estimate for this type of synaesthesia, not based on self-report, of 0.2% by Simner et al. (2006).

## Discussion

There is convincing evidence for a relationship between synaesthesia and engagement in the visual arts, but the evidence with regard to music is sparser and mixed. For example, Rich et al. (2005) did not find that synaesthetes were more likely to be actively engaged in music as a hobby and did not report it as one of their strengths. Other studies have pointed to greater engagement in music by synaesthetes (Lunke & Meier, 2022; Ward et al., 2008), albeit with a qualification that the effect is particularly pronounced in certain synaesthetes (e.g., those synaesthetes for whom music, and other sounds, elicit visual experiences). The present study takes a somewhat different approach of investigating the prevalence of synaesthesia in musicians considering grapheme-colour, sequence-space, and music tone – colour. There are far fewer prevalence estimates for the latter owing to a lack of well-validated methods for this type (i.e., based on an optimised cut-off between synaesthetes and non-synaesthetes), and we use a recently published approach (Ward et al., 2024). In brief, we find an increased prevalence of all three kinds of synaesthesia amongst musicians. In a secondary analysis, we show that synaesthesia is particularly prevalent in people who engage, at a high level (supplementing their income), in multiple creative pursuits (i.e., music + art) relative to one or none of these domains.

The fact that sequence-space synaesthesia (for days, months, and numbers) is over-represented in musicians may seem surprising given that it is not directly relevant to music, although it could be that these individuals also have spatial representations of musical structure (sequences of notes, chords, scales, finger positions) that we did not assess. It also speaks against the possibility that they could simply have used their musical skills (e.g., in pitch perception) to merely appear like a synaesthete. It suggests instead that there are skills and traits within most, if not all, types of synaesthesia that facilitate engagement and success in music. This has sometimes been referred to as a synaesthetic disposition (Ward, 2019) or a general synaesthetic trait (Rouw & Scholte, 2016). These differences might include greater mental imagery across multiple senses, greater attention-to-detail, and higher openness to experience. Of course, these traits are not unique to synaesthetes but might be over-represented in this group. There is also evidence that heterogeneous groups of synaesthetes (not just those with sound/music inducers) perform better in tests of auditory perception such as detecting a tone in noise (Del Rio et al., 2024) and in memory for musical phrases (Mealor et al., 2020). It may still be the case that those individuals with music-colour synaesthesia have important differences relative to other synaesthetes in measures that we have not been able to capture here (e.g., in pitch or rhythm perception). Previous research suggests that these music-colour synaesthetes gravitate to certain music genres that are musically complex and layered (Ward et al., 2024). It may also be that they occupy different roles within a band or orchestra (e.g., leading or composing versus following), are more commercially successful, or more able to detect nuances missed by others (e.g., how a current performance differs from a previous one). The latter could be directly aided by being able to ‘see’ the music. These would be important measures to collect in future research.

There is a paucity of research on the prevalence of sound-colour synaesthesia, but it is generally considered to be rarer than grapheme-colour synaesthesia in both self-selected samples of synaesthetes (Ward & Simner, 2022) and from screening of opportunistic samples in the general population (Simner et al., 2006). Here we do not find that to be the case which requires further discussion (in non-musicians the prevalence of sound-colour was 1.3% and that of grapheme-colour was 0.2%). Firstly, our approach for classifying a person as having this type of synaesthesia does not rely solely on consistency but takes into account other measures (e.g., the overall palette of colours). This was necessitated by the fact that consistency is a less reliable discriminator for this type than others. Rather than a simple pass-fail, one could interpret this as different degrees of evidence for having this type of synaesthesia. Importantly, the finding of increased prevalence in musicians is robust against this. Whilst it is also conceivable that some participants are false positives (i.e., do not have synaesthesia), we minimised this by only sending the tests to participants who indicated that they had these kinds of experiences in the first place. It would be important for future research to compare this with study designs in which everyone takes the same tests (whether reporting synaesthesia or not) so that test performance and self-report can be considered separately. Another possibility for why we find sound-colour synaesthesia to be more prevalent than grapheme-colour could be that our non-musician control group are not truly representative of the general population (i.e., they contain more people engaged in music than might be expected perhaps at a hobby rather than semi-professional level). The criteria for being classed as a ‘musician’ can also be called in question and could, in theory, lead to different prevalence results. However, we show that our results are broadly robust against the three different classification systems considered here (see again Figure 1): being part of a professional music organisation (strongest criteria), earning money from music, or self-identifying as a musician (weakest criteria). Future research might consider augmenting these results with validated measures of musicality (e.g., Müllensiefen et al., 2014).

In conclusion, we provide convincing evidence that synaesthesia, in various forms, is more prevalent amongst musicians. We explain this in terms of synaesthesia being linked to cognitive abilities that are conducive to musical talent, as well as motivational factors (e.g., openness to experience) that encourage engagement with creative pursuits.
